# Patient-centered factors associated with orthodontic treatment success: a scoping review

**DOI:** 10.1590/1807-3107bor-2025.vol39.020

**Published:** 2025-02-21

**Authors:** Lucas Garcia Santana, Luisa Gatti-Reis, Saul Martins Paiva, Maria Letícia Ramos-Jorge, Leandro Silva Marques

**Affiliations:** (a) Universidade Federal dos Vales do Jequitinhonha e Mucuri – UFVJM, Department of Pediatric Dentistry and Orthodontics, Diamantina, MG, Brazil.; (b) Universidade Federal de Minas Gerais – UFMG, Department of Pediatric Dentistry, Belo Horizonte, MG, Brazil.

**Keywords:** Patient-Centered Care, Review, Evidence-Based Practice, Orthodontics

## Abstract

The aim of this systematic review was to synthesize the available literature on pretreatment factors, from a patient-centered perspective, that predict successful orthodontic treatment. Systematic and unrestricted searches were conducted across the electronic MEDLINE, Embase, Web-of-Science, Scopus, Cochrane Library, and LILACS/Bireme databases. Grey literature was also searched to identify potential studies. Qualitative assessments of the role of patient-centered pretreatment factors in orthodontic treatment success (adherence, satisfaction, and discontinuation rates) were evaluated and synthesized. Study selection and data extraction were performed independently by two reviewers. A total of sixteen studies were included. Three main domains related to the patient emerged as positive predictors of orthodontic treatment success: previous patterns of social behavior, attitudes of parents/caregivers, and reasonable motivation. Certain personality traits were associated with both positive and negative levels of adherence and treatment satisfaction. The evidence did not support the notion that the impact of malocclusion on aesthetic self-perception could serve as an indicator of cooperation, satisfaction, or discontinuation rates related to orthodontic treatment. Positive social behavior patterns and parental/caregiver attitudes, along with realistic prior motivation toward treatment goals, were found to be reliable predictors of orthodontic treatment success. Additionally, some personality traits were found to be associated with successful treatment outcomes. However, the evidence did not support the impact of malocclusion on self-perceived aesthetics as an indicator of compliance and satisfaction levels with orthodontic treatment.

## Introduction

The success of orthodontic treatment has traditionally been assessed using morphological outcomes and long-term stability rates.^1^ Although clinical assessment of malocclusion and knowledge of techniques are essential for orthodontists adopting an evidence-based approach to treatment, it is also crucial to consider patients’ values and experiences.^1,2^ Previous studies have highlighted that patient-centered factors, such as the psychosocial impact of malocclusions,^3,4^ motivations, expectations regarding orthodontic treatment,^5,6^ and certain personality traits, may influence the success of orthodontic treatment. In 2015, a systematic review showed that patient satisfaction following treatment is associated with aesthetic results, psychological benefits, and the quality of care provided by the clinician and staff. ^5,6^

Understanding how patients perceive their treatment is essential for accurate diagnosis and optimized clinical decision-making.^2^ This raises the question of whether there is a reliable way for clinicians to identify potentially “difficult” patients.^7^ Furthermore, developing a core set of patient-centered pretreatment traits that could assist in clinical management and communication/relationship strategies based on these characteristics would be valuable.^2^ Orthodontists could use this information, alongside objective diagnostic criteria, to enhance evidence-based practice. Given the potential relationship between pretreatment patient-centered factors and orthodontic treatment success, it is important to understand how these factors influence outcomes.^1^

Scoping reviews aim to address an exploratory research question by mapping concepts, existing evidence, and identifying relevant questions for future studies.^7^ As a form of knowledge synthesis, scoping reviews employ robust, transparent, and reproducible methods.^8^ This review aimed to assess the literature on patient-centered factors related to successful orthodontic treatment. A scoping review was deemed most appropriate, as it allows for mapping an extensive topic and can be used to highlight relevant characteristics that may be associated with a concept.^8^

## Methods

The study was conducted following the PRISMA Extension for Scoping Reviews (PRISMA-ScR), namely the Preferred Reporting Items for Systematic Reviews and Meta-Analyses Extension for Scoping Reviews.^8^

### Eligibility criteria

The research question was: What patient-centered factors (subjective and objective) are related to successful orthodontic treatment? The question was based on the PCC acronym—Population, Concept, Context—where P refers to orthodontic patients of any age from private clinics, hospitals, or universities; C refers to successful orthodontic treatment, including levels of adherence, treatment completion (discontinuation rate), and/or satisfaction/experiences with orthodontic treatment; and C refers to subjective and objective patient-centered factors, such as malocclusion, patient expectations, personality traits, self-perception, oral health-related quality of life, psychosocial factors, or other characteristics described by the authors.

Randomized and non-randomized controlled trials, as well as observational and qualitative studies exploring patients’ values and experiences, were considered eligible for inclusion. Reviews, editorials/letters, studies evaluating the clinical outcomes of techniques, studies involving orthognathic surgery, and studies with participants having craniofacial syndromes were excluded. All model development and validation studies (both internal and external) were included.

### Information sources and search strategy

A comprehensive search of the MEDLINE (PubMed), Embase, Web-of-Science, Scopus, Cochrane Library, and LILACS/Bireme databases was conducted in October 2022 and updated in September 2024. The search strategy is presented in Table 1. No restrictions on language, publication year, or publication status were applied. A gray literature search was performed using Google Scholar, and manual searches were conducted in the reference lists of the included articles.

**Table 1:** Search strategy.

Electronic database	Search strategy used	Items found
MedLine	(((orthodontics) OR (orthodontics treatment)) AND (((((((((quality of life)) OR (patient-experiences)) OR (patient-concerns)) OR (patient-expectation)) OR (treatment expectations)) OR (patient- self-perception)) OR (impact of malocclusion)) OR (patient compliance)))) AND ((((treatment success) OR (success rate)) OR (discontinuation of treatment)) OR (patient satisfaction))	927
Searched via PubMed on October 2022; Update on September 2024		
Embase	#1 ‘orthodontics’:ti,ab,kw OR ‘orthodontic treatment’:ti,ab,kw	76
Searched via PubMed on October 1, 2022; Update on September 2024	#2 ‘quality of life’:ti,ab,kw OR ‘patient experiences’:ti,ab,kw OR ‘patient concerns’:ti,ab,kw OR ‘patient expectation’:ti,ab,kw OR ‘treatment expectations’:ti,ab,kw OR ‘patient- self-perception’:ti,ab,kw OR ‘impact of malocclusion’:ti,ab,kw OR ‘patient compliance’:ti,ab,kw	
	#3 ‘treatment success’:ti,ab,kw OR ‘success rate’:ti,ab,kw OR ‘discontinuation of treatment’:ti,ab,kw OR ‘patient satisfaction’:ti,ab,kw	
	#1 AND #2 AND #3	
Web of Science	#1 TOPIC: (orthodontics) OR TOPIC: (orthodontic treatment)	360
Searched on October 1, 2022; Update on September 2024	#2 TOPIC: (quality of life) OR TOPIC: (patient-experiences) OR TOPIC: (patient-concerns) OR TOPIC: (patient-expectation) OR TOPIC: (treatment expectations) OR TOPIC: (patient- self-perception) OR TOPIC: (impact of malocclusion) OR TOPIC: (patient compliance)	
	#3 TOPIC: (treatment success) OR TOPIC: (success rate) OR TOPIC: (discontinuation of treatment) OR TOPIC: (patient satisfaction)	
	#1 AND #2 AND #3	
Cochrane Central Register of Controlled Trials	#1 (orthodontics):ti,ab,kw OR (orthodontic treatment):ti,ab,kw	48
Searched on October 1, 2022; Update on September 2024	#2 (“quality of life” OR “patient-experiences” OR “patient-concerns” OR “patient-expectation” OR “treatment expectations” OR “patient- self-perception” OR “impact of malocclusion” OR “patient compliance”):ti,ab,kw	
	#3 (treatment success):ti,ab,kw OR (success rate):ti,ab,kw OR (discontinuation of treatment):ti,ab,kw OR (patient satisfaction):ti,ab,kw	
	#1 AND #2 AND #3	
Scopus	#1 ( TITLE-ABS-KEY ( orthodontics ) OR TITLE-ABS-KEY ( orthodontic AND treatment ) )	545
Searched on October 1, 2022; Update on September 2024	#2 ( TITLE-ABS-KEY ( quality AND of AND life ) OR TITLE-ABS-KEY ( patient-experiences ) OR TITLE-ABS-KEY ( patient-concerns ) OR TITLE-ABS-KEY ( patient-expectation ) OR TITLE-ABS-KEY ( treatment AND expectations ) OR TITLE-ABS-KEY ( patient- AND self-perception ) OR TITLE-ABS-KEY ( impact AND of AND malocclusion ) OR TITLE-ABS-KEY ( patient AND compliance ) )	
	#3 ( TITLE-ABS-KEY ( treatment AND success ) OR TITLE-ABS-KEY ( success AND rate ) OR TITLE-ABS-KEY ( discontinuation AND of AND treatment ) OR TITLE-ABS-KEY ( patient AND satisfaction ) )	
	#1 AND #2 AND #3	
LILACS database	((orthodontics) OR (orthodontic treatment)) AND ((quality of life) OR (patient-experiences) OR (patient-concerns) OR (patient-expectation) OR (treatment expectations) OR (patient- self-perception) OR (impact of malocclusion) OR (patient compliance)) AND ((treatment success) OR (success rate) OR (discontinuation of treatment) OR (patient satisfaction))	62
Searched October 1, 2022; Update on September 2024		
Google Scholar	((orthodontics) OR (orthodontic treatment)) AND ((quality of life) OR (patient-experiences) OR (patient-concerns) OR (patient-expectation) OR (treatment expectations) OR (patient- self-perception) OR (impact of malocclusion) OR (patient compliance)) AND ((treatment success) OR (success rate) OR (discontinuation of treatment) OR (patient satisfaction))	0
Searched October 1, 2022; Update on September 2024		
Manual Search		11
Sum		2029

### Study selection, data items and collection

Two authors (LGS, LSM) independently screened the titles and abstracts of the references. References potentially eligible for inclusion were retrieved for full-text analysis. The same authors independently assessed the full text of the studies, and those meeting the eligibility criteria were included. Reasons for excluding studies at this phase were documented (Table 2).. In both phases, disagreements were resolved by consensus.

**Table 2:** Number of excluded studies and reasons for exclusion after full-text evaluation.

Reasons for exclusion	Number of studies
Assessed occlusal outcomes	4
Book chapter	1
Commentary/editorial	3
Description of clinical technique	3
Did not assess patient-centered predictive factors	20
Did not evaluate outcome success	45
Reviews	8
Sample included individuals with syndromes or who underwent orthognathic surgery	6
Sum	90

Data extraction was conducted independently by the two reviewers (LGS, LSM). An extraction template was developed to outline the characteristics of interest and guide the reviewers. The template included the following information: study identification, settings, participants, measurements, outcomes, evaluation period (timing), and main findings. The data obtained from each reviewer were compared for accuracy, and any discrepancies were resolved by reexamining the original study.

### Synthesis of results

The data from qualitative studies were synthesized by themes in a descriptive table, followed by the integration of results. Heterogeneity in the assessment of outcomes across the included studies was considered prior to making comparisons.

## Results

### Study selection

The electronic search strategy yielded 2,018 references, and 11 were identified through manual searches (Figure). After duplicates were removed and titles/abstracts screened, 114 studies were retrieved for full-text evaluation. Of these, 103 were excluded, with the reasons provided in a flowchart. Ultimately, 16 studies met the eligibility criteria and were included.

**Figure:**
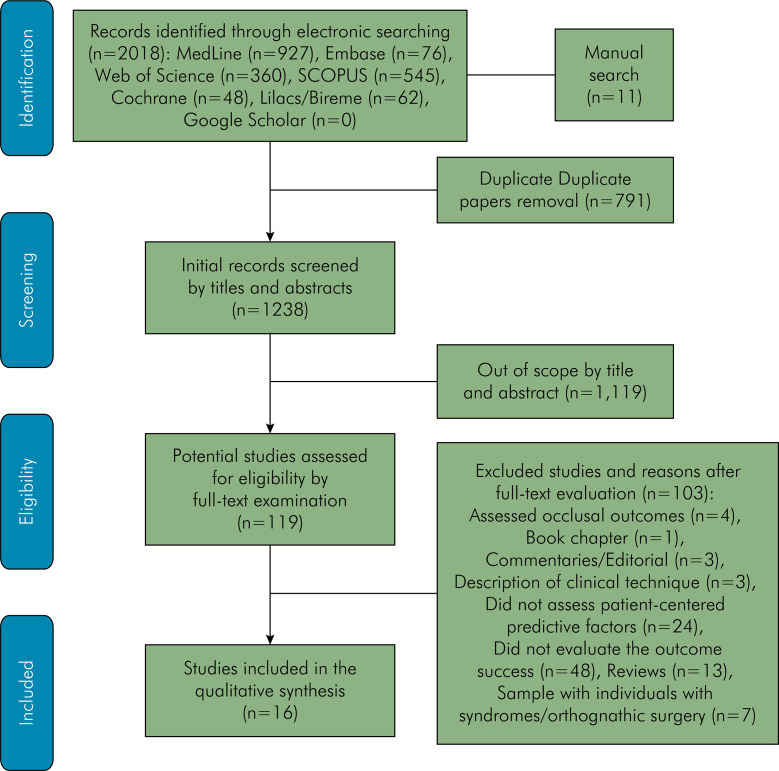
PRISMA flow diagram illustrating the study selection process.

### Study characteristics

Table 2 presents the descriptive characteristics of the included studies. Approximately 52% of the studies were published within the past 10 years. Samples were drawn from various countries, including Colombia,^9^ Croatia,^10^ Germany,^11-13^ Iran,^14^ Jordan,^15^ New Zealand,^16^ Poland,^17,18^ South Korea,^19^ Sweden,^20^ the United Kingdom,^3,21^ and the United States of America^5,22^. Participants were from colleges/universities,^5,10-13,15,17-20,22^ hospitals^3,16^, and private clinics.^14^ A total of 2,060 participants were enrolled, with sample sizes ranging from 38,^18^ to 298^19^ individuals. The mean age of participants at baseline ranged from 10^13,14^ to 20.7^15^ years (age range: 9^13,17,18^ to 67^19^ years). Various orthodontic treatment approaches were identified, including removable functional appliances,^3,10,12-14,17,18^ multibracket fixed appliances,^3,11,15,20^ and elastic/headgear.^22^ Five studies did not specify the type of treatment involved.^5,9,16,19,21^

Treatment success was determined based on satisfaction with treatment (6 studies^5,14-16,19,20^), adherence (9 studies^9-13,17,18,21,22^), and discontinuation rate (2 studies^3,10^). Fourteen studies used questionnaires as assessment tools, covering personality/emotional traits,^9,10,14,15,17,22^ the impact of malocclusion on patient perception,^3,17^ motivation for treatment,^5,13,19,20,22^ social behavior,^16,21,22^ and satisfaction.^14,15,20^ Of these, one study^17^ employed a non-validated questionnaire, while 4 studies^13,19,21,22^ used customized questionnaires. Four studies^5,10,18,21^ administered questionnaires to both patients and their caregivers simultaneously. Other factors, such as pretreatment occlusal features^3,11,14,15,20^ and the patient’s level of cooperation according to body mass index^11,12^ were assessed in some of the included studies.

### Synthesis of the studies

Qualitative comparisons were conducted (Tables 3 and 4).

**Table 3:** Summary of characteristics and outcome measurements of the studies included.

Study ID	Participants and settings	Outcomes and/or measurement method	Evaluation period	Main findings
Stefanovic et al., 2021^10^	n = 77 (36 F, 41 M)	Psychosocial assessments.	1 y	Parental perception of altered emotional well-being of their children was the strongest predictor, increasing compliance odds by 3.4 times (95% CI, 1.2–9.4; p = .017).
	Mean age: 12 y	Discontinued treatment.		Patients with overjet > 8 mm were 3.1 times more compliant (95% CI, 1.0–9.4; p = .044).
	Age range: 11–13 y	Compliance.		Children’s self-assessed oral health and teeth appearance satisfaction were not valid predictors (p > 0.05).
	Treatment: removable functional appliances			
	Location: University Dental Clinic, Rijeka, Croatia			
Naseri et al., 2020^14^	n = 50 (29 F, 21 M)	Personality traits.	3 mo	A correlation was found between self-efficacy, total score in the acceptance questionnaire (r = 0.486, p = 0.001), and level of liking of the orthodontic appliance (r = 0.530, p = 0.001).
	Mean age: 10.5 y	Severity of malocclusion.		There was no relationship between IOTN and the patient’s level of acceptance (p > 0.05).
	Age range: 10–12 y	Satisfaction.		
	Treatment: removable orthodontics			
	Location: private clinic, Shiraz, Iran			
Sarul et al., 2019^17^	n = 97 (51 F, 46 M)	Self-perception of smile attractiveness.	9 mo	DWT of participants with low self-perception of smile attractiveness: mean 9.64 +- 2.77 h.
	Age range = 9–12 y	Compliance (DWT measurements with TheraMon® software).		DWT of participants with low self-perception of smile attractiveness: mean 5.92 +- 2.28 h.
	Treatment: removable functional appliances			(p < 0.001)
	Location: Wroclaw Medical University, Wroclaw, Poland			There was a strong negative correlation between DWT and poor smile as regarded by patients (r = −0.77).
Lee et al., 2018^19^	n = 298 (207 F, 91 M)	Satisfaction.	NR	Treatment satisfaction was positively related to motivation; the level of satisfaction with tooth alignment (92%), confident smile and self-image (71%) were higher than that with facial appearance (60%) and eating and chewing (59%) (p < 0.001).
	Age range: 21–67 y			Concerns with having to attend multiple regular visits and discomfort due to an inconvenient hospital system negatively influenced the level of satisfaction (p < 0.05).
	Treatment: NR			
	Location: Yonsei University, Seoul, South Korea			
Von Bremen et al., 2018^12^	n = 114	Compliance (DWT measurements with TheraMon® software).	6 mo	An indirect association between BMI and appliance wear time, indicating that the higher the BMI, the less the patients wore their appliances (r = -0.267, p < 0.05).
	Mean age: 11 y			
	Treatment: removable appliances			
	Location: University of Giessen, Giessen, Germany			
Sarul et al, 2017^18^	n = 38 (19 F, 19 M)	Personality and parental traits.	9 mo	There was a strong negative correlation between DWT in children classified as having an emotional temperament, with a tendency to experience emotions intensely (r = -0.54, p < 0.01).
	Age range = 9 - 12 y	Compliance (DWT measurements with TheraMon® software).		The severity of the requirements imposed on the child (r = 0.591, p < 0.001), the child’s sense of self-efficacy (r = 0.511, p = 0.001) and conscientiousness (r = 0.721, p < 0.001) of the parents were positively correlated with the patient’s cooperation.
	Treatment: removable functional appliances			
	Location: Wroclaw Medical University, Wroclaw, Poland			
Von Bremen et al., 2016^11^	n = 175 (88 F, 87 M)	Compliance	COT	There was a tendency for an increased BMI to appear as a risk factor for less cooperation (48% of normal weight patients and 20% of obese patients cooperated sufficiently), although this was not statistically significant (p = 0.16).
	Mean age: 12.9 y			
	Treatment: multibracket fixed appliance			
	Location: University of Giessen, Giessen, Germany			
Feldmann, 2014^20^	n = 110 (56 F, 54 M)	Motivation.	COT (mean = 25.3 mo)	There was a tendency toward significant correlations between prior treatment motivation and overall satisfaction with treatment (p < 0.01).
	Mean age: 16.9 y	Expectations.		The patient’s own decision to start treatment and the PAR index pre- and posttreatment did not correlate with treatment satisfaction (p > 0.05).
	Treatment: multibracket fixed appliance	Satisfaction.		
	Location: Public Dental Service, Gvleborg County Council, Gävle, Sweden.	Severity of malocclusion.		
Anderson et al., 2009^5^	n = 147	Personality and parental traits	COT	Patients more focused on the post-treatment esthetics (r = 0.337, p = 0.004) and functioning (r = 0.231, p = 0.053;) outcomes and more energized by thinking about their posttreatment possible selves were more satisfied with the treatment.
	Mean age: 11.6 y			Parents were also more satisfied with the treatment when they believed that their children were energized by thinking about their posttreatment possible selves (r = 0.326, p = 0.007).
	Treatment: NR			
	Location: University of Michigan, USA			
Amado et al., 2008^9^	n = 70 (46 F, 24 M)	Personality traits.	At least 4 mo	The patient’s personality traits of extroversion/introversion, self-control and harshness do not predict cooperation during the orthodontic treatment (p < 0.05).
	Mean age: 13.4 y	Compliance.		
	Age range: 12–15 y			
	Treatment: NR			
	Location: CES University, Medellin, Colombia			
Mandall et al., 2008^3^	n = 144 (79 F, 65 M)	Discontinued treatment.	COT (mean = 16.6 mo)	The IOTN score and the aesthetic impact of malocclusion variables showed no association with completion of orthodontic treatment (logistic regression analysis; p> 0.05).
	Mean age: 13.7 y	Severity of malocclusion.		
	Age range: 10 – 19 y			
	Treatment: fixed or removable appliances			
	Location: University Dental Hospital of Manchester, Bolton Royal Hospital, St Anne’s Orthodontic Practice, and Hope Hospital, Salford, UK			
Al-Omiri et al., 2006^15^	n = 50 (30 F, 20 M)	Personality traits.	COT (mean, 19 mo)	Patients with high neuroticism scores were associated with lower levels of satisfaction with the dentition (r^2^ = -0.367, p < .01). Other personality traits (extroversion, openness, agreeableness, conscientiousness) demonstrated no relationship with satisfaction (P > 0.05).
	Mean age: 20.7 y	Satisfaction.		The IOTN had no relationship with the patient’s satisfaction (p > 0.05).
	Age range: 13–28 y	Severity of malocclusion.		
	Treatment: multibracket fixed appliance			
	Location: Jordan University of Science and Technology, Irbid, Jordan			
Barker et al., 2005^16^	n = 294	Personality traits.	NR	Individuals with lower scores of social closeness were twice as likely to be dissatisfied with their orthodontic result (OR: 2.07, p = 0.02).
	Age range: 15 - 26 y	Satisfaction.		The DAI had no relationship with the patient’s satisfaction (p > 0.05).
	Treatment: any type of orthodontic appliance	Severity of malocclusion.		
	Location: Queen Mary Hospital, Dunedin, New Zealand.			
Bartsch et al., 1993^1^	n = 77 (37 F, 40 M)	Compliance (DWT measurements with TheraMon® software).	3.9 mo	Both internal motivation and parental involvement were beneficial for compliance (r = 0.41, p < 0.001; r = 0.50, p < 0.001).
	Mean age: 10.2 y	Psychosocial assessments.		Compliant patients are more often exposed to positive social models (r = 0.57, p <0.001).
	Age range: 9–15 y	Motivation.		Compliant patients report a high need for academic achievement (r = 0.36, p < 0.01).
	Treatment: removable functional appliances			
	Location: Würzburg university, Würzburg, Germany			
Egolf et al., 1990^22^	n = 100	Personality traits.	at least 3 mo under treatment	Four factors were found to correlate weakly, but significantly,
	Mean age: 15.3 y	Compliance.		with compliance: internal/external motivation for treatment (r = 0.241, p = 0.017), health awareness (r = -0.289, p = 0.004), stoic/sensitive personality (r = -0.374, p < 0.001), and self-confidence (r = 0.252, p = 0.012).
	Treatment: headgear and intraoral elastics	Motivation.		The importance of straight teeth, oral beauty, and the social importance of beauty were not correlated with compliance (p > 0.05).
	Location: University of Illinois College of Dentistry, Chicago, USA			
Woolass et al., 1988^21^	n = 219	Personality traits.	3 y	The best cooperation group reported an increased self-concept of behavior (MD = 9.01, p <0.01), reported being able to hide their emotions (MD = 1.73, p < 0.001), and were more popular (MD = 0.68, P < 0.01), more sociable (MD = 0.60, P < 0.01) and more confident (MD = 0.49, p < 0.02).
	Age range = 11–12 y	Psychosocial assessments.		Patients with antisocial behavior were associated with poor compliance (MD = 0.71, p < 0.05).
	Treatment: NR	Compliance.		
	Location: Cardiff, UK			

BMI: body mass index; COT: Completed orthodontic treatment; DAI: dental aesthetic index; DWT: daily wear time; EAS-C: emotionality activity sociability-children; F: female; h: hours; IOTN: index of orthodontic treatment need; M: male; mo: months; NR: not reported; PAR: peer assessment rating; y: years.

**Table 4:** Characteristics positively associated, negatively associated, and with no association with successful treatment outcomes.

Variable	Personality traits	Impact of malocclusion	Previous motivation	Social behavior	Caregiver factors	Other
Positive association						
Compliance	Self-confidence	NR	- Internal motivation	Positive behavior models	Caregiver motivation	Severity of malocclusion
	Health			Good social interactions	Conscientiousness	
	Awareness			Self-efficacy		
	Stoic/sensitive personality			High academic achievement		
	Emotional control					
Satisfaction	Focused	NR	- Internal motivation	Positive behavior models	Caregiver motivation	NR
			- Realistic motivation	Good social interactions	Conscientiousness	
				- Self-efficacy;		
				- High academic achievement.		
Discontinuation rate	NR	NR	NR		NR	NR
Negative association						
Compliance	Emotionally intense	NR	NR	Antisocial behavior	-	Increased body mass index
Satisfaction	Neuroticism	NR	NR	Low social closeness	-	Multiple regular appointments
Discontinuation rate	NR	NR	NR	NR	-	NR
No association						
Compliance	NR	Self-assessed teeth appearance	NR	NR	NR	NR
		Impact of teeth and oral appearance on social interactions				
Satisfaction	Extroversion	NR	NR	NR	NR	Severity of malocclusion
	Harshness					Own decision to start treatment
	Openness					Hospital/clinical system
	Agreeableness					
	Conscientiousness					
Discontinuation rate	NR	Aesthetic impact of malocclusion	NR	NR	NR	Severity of malocclusion

NR: not reported.

#### Personality traits associated with treatment success

Several personality traits were linked to successful treatment from a patient-centered perspective. In terms of subjective analysis of treatment adherence, individuals who were more self-confident,^21,22^ had health awareness,^22^ and displayed a stoic or sensitive personality^22^ were more likely to cooperate by wearing the appliance, avoiding breakage, attending appointments, and maintaining good oral hygiene. Two studies^18,21^ evaluated the association between emotional expression and cooperation. Individuals who reported being able to control their emotions were associated with better cooperation.^21^ Similarly, individuals with a tendency to experience emotions intensely were negatively correlated with the objective measurement of appliance daily wear time (r = –0.54, p < 0.01).^18^ Regarding satisfaction with treatment, patients who were more energized and focused prior to treatment were generally more satisfied with the treatment outcome.^5^ However, patients with high neuroticism scores were associated with dissatisfaction (r = –0.367, p < 0.01).^15^ Personality traits such as extroversion, harshness, openness, agreeableness, and conscientiousness showed no significant association with satisfaction.^9,15^

#### Impact of initial malocclusion

Four studies^3,10,17,20^ analyzed the impact of initial malocclusion on individuals through self-perceived smile or oral esthetics. The results indicated that this variable was generally an unreliable predictor of treatment success. One study^3^ found that the impact of malocclusion was not statistically associated with the completion rate of orthodontic treatment. Cooperation was assessed in three studies, and factors such as self-assessed teeth appearance^10^ and the impact of straight teeth and oral beauty on social interactions^22^ were not associated with a subjective analysis of adherence. Only one study, with a high risk of bias,^17^ reported a negative correlation between appliance daily wear time and poor smile perception as regarded by patients.

#### Motivation to undergo treatment

Prior motivation and expectations appear to be key factors, as all the studies^5,13,19,20,22^ that analyzed this variable identified it as an influencing factor in the success of orthodontic therapy. Two studies^13,22^ reported that internal motivation was beneficial for adherence, whether cooperation was assessed objectively, using a microelectronic timing system^13^ for appliance wear, or subjectively by the clinician.^22^ Similarly, treatment satisfaction was positively related to motivation before treatment.^5,19,20^ The more realistic and consistent a patient’s previous expectations were with the treatment goals, the more satisfied they were with the outcome.^5,19^ Notably, adult patients’ satisfaction with tooth alignment (90%), a confident smile, and self-image (71%) was higher than their satisfaction with facial appearance (60%).^19^

#### Social behavior

The four studies^13,14,16,21^ that analyzed the social behavior of patients identified similar key themes associated with the outcome of orthodontic treatment. Individuals who were more frequently exposed to positive social models,^13^ including better social interactions,^13,21^ self-efficacy behaviors,^14^ and a high need for academic achievement,^13^ were more likely to experience successful orthodontic treatment in terms of both adherence and satisfaction. Conversely, individuals displaying antisocial behavior were associated with poor adherence to treatment,^21^ while those with lower scores for social closeness were twice as likely to be dissatisfied with their orthodontic therapy (OR: 2.07, p = 0.02).^16^

#### Parental/caregivers

The role of parents and caregivers appears to have a significant influence on children’s cooperation with treatment.^10,13,18^ The parents’ perception of the impact on their children’s emotional well-being during treatment increased adherence by 3.4 times (p = 0.017).^10^ Additionally, the internal motivation of parents,^5,13^ the perceived sense of self-efficacy in their children,^18^ and the parents’ conscientiousness^18^ were positively correlated with patient cooperation and/or satisfaction.

#### Other factors

Some studies evaluated morphological characteristics alongside psychosocial aspects. The severity of malocclusion, assessed through indices of the need for orthodontic treatment, was not associated with treatment discontinuation^3^ or patient satisfaction rates.^14-16,20^ One study^10^ found that patients with a greater overjet (> 8 mm) were 3.1 times more compliant with removable appliances. However, this result should be interpreted with caution, as the method used to assess adherence included the clinical outcome of treatment, which can be influenced by other variables.

Finally, other factors assessed included the patient’s own decision to start treatment, which was not associated with satisfaction^20^, and the need to attend multiple regular visits and discomfort related to an inconvenient hospital system, which were associated with treatment outcome dissatisfaction.^19^ Two studies^11,12^ examined whether there is a correlation between body mass index (BMI) and patient cooperation. Subjective analysis found that an increased BMI appears to be a risk factor for poorer cooperation,^11^ which was confirmed when appliance daily wear time was measured with microsensors (r = –0.267, p < 0.05).^12^

## Discussion

### Summary of evidence

Three main dimensions related to the patient were found to be predictors of the potential success of orthodontic treatment: patterns of social behavior, parental/caregiver attitudes, and prior motivation. The evidence supported that certain personality traits were associated with successful treatment outcomes. However, the evidence did not support that the impact of malocclusion on esthetic self-perception could be an indicator of levels of cooperation and satisfaction with orthodontic treatment.

One previous hypothesis was that the severity of the malocclusion and its impact on psychosocial factors would predict treatment adherence, as it is well established that occlusal discrepancies negatively affect the lives of schoolchildren, particularly in social and psychological contexts.^23,24^ However, most of the included studies that assessed this variable did not find this association. In general, individuals with greater sociability, more intense social interactions, and high academic performance are predictive of higher levels of compliance and satisfaction with treatment.^13,21^ Based on this evidence, factors such as family and school environment, lifestyle, and the influence of parents may have a greater impact on the social aspects of quality of life than malocclusion alone. This underscores the importance of considering how well we understand the orthodontic patient at both the individual and social levels to ultimately improve patient adherence and satisfaction.^1^

As mentioned earlier, the severity of malocclusion was not significantly associated with the level of satisfaction. This suggests that individuals seeking orthodontic treatment for relatively minor malocclusions have a similar likelihood of being dissatisfied with their orthodontic results as those who are more severely affected before treatment.^9,14-16,20^ It is noteworthy that treatment for dental misalignment, although less complex from a morphological standpoint, can be just as challenging as severe malocclusion in achieving treatment success from a perspective that reflects the patient’s values.

The pattern of social behavior, as well as parental attitudes towards children’s education, can be important predictors of the effectiveness of orthodontic therapy. The term self-efficacy, which refers to the belief in one’s ability to meet established goals, appears to be a key variable in forming health behaviors and fostering cooperation in the treatment of diseases.^18^ Determinants of the effectiveness of therapy and the prevention of dental diseases in children suggest that both children’s and parents’ sense of self-efficacy regarding their child has a significant positive effect.^25^ This also applies to orthodontic therapy, where a high sense of efficacy was associated with greater cooperation and satisfaction with treatment.^14,18^

### Limitations and strengths

The main sources of heterogeneity in this scoping review were related to the data, the variety of instruments used, and the recruitment of comparable groups. It should be noted that this is particularly challenging in this type of research. Furthermore, the review was not conducted to compare techniques or treatments but to assess qualitative information. As such, the inclusion of observational study designs was deemed appropriate.

Most of the included studies used a convenience sample, which limits the generalizability of the results. However, it is noteworthy that the studies were conducted in several countries across different regions, which helps minimize potential bias from local factors. A possible selection bias was addressed by conducting extensive searches across multiple electronic databases, including the search for unpublished and ongoing studies on clinical trial registration platforms, and by assessing partial gray literature without restrictions on language or publication status.

### Implications for practice and research

Personalized healthcare not only addresses the effectiveness of therapies but also focuses on how to use an individual’s information to make tailored decisions about the most appropriate treatment.^26^ This review provides evidence on potential patient-centered factors associated with successful orthodontic treatment. Poor adherence to therapies could compromise treatment effectiveness, highlighting this critical issue from both occlusal traits and quality of life perspectives. While assessing the effectiveness of techniques is necessary, as clinicians need to evaluate the interventions they will perform, the results of this study are also relevant. They provide essential information to assist practitioners in making clinical decisions, such as choosing between a fixed functional appliance or skeletal anchorage for patients identified as potentially having poor adherence. Additionally, identifying patients with potentially negative psychosocial traits enables more appropriate management of the dentist-staff-patient relationship, which can prevent or reduce future dissatisfaction.

In this review, motivation and expectations related to undergoing orthodontic treatment were strongly linked to treatment adherence and patient satisfaction. The available evidence supports the benefits of orthodontic treatment in reducing the risk of periodontal damage, caries development, or TMJ disorders. However, the science is clear that these potential benefits have limitations.^27^ Additionally, it should be noted that most patients seek orthodontic treatment for esthetic reasons, and achieving the desired smile may improve their social life and help them make a good impression.^28^ Furthermore, the use of visual social media has been reported to create unrealistic expectations of an ideal smile, increasing facial and smile dissatisfaction when a person’s ‘current’ self does not match their ‘ideal’ self.^29,30^ With the increasing use of social media, clinicians must be aware of its impact on patients. It is essential to determine whether patients’ initial expectations are realistic and aligned with the anticipated treatment outcomes. Further studies should assess the impact of social media on the compliance, satisfaction, psychosocial factors, and mental well-being of orthodontic patients. In this context, the variables discussed herein can serve as a foundation for future studies evaluating the role of patient-centered outcomes in the orthodontic field. Ultimately, understanding a patient’s psychosocial aspects must be integrated into the diagnostic process, alongside the normative aspects of malocclusion. Only then can we provide the highest quality, patient-centered care.

## Conclusions

A positive pattern of social behavior, parental/caregiver attitude, and realistic expectations regarding orthodontic treatment can be reliable predictors of adherence and satisfaction.

Personality traits appear to be reliable predictors for both positive and negative levels of adherence and satisfaction.

The psychosocial impact of initial malocclusion, self-perception of one’s smile, and the severity of malocclusion were not strongly associated with adherence, satisfaction, or treatment discontinuation rates.
